# Triterpenoids from the Roots of *Sanguisorba tenuifolia* var. Alba

**DOI:** 10.3390/molecules16064642

**Published:** 2011-06-03

**Authors:** Hai-Xue Kuang, Hong-Wei Li, Qiu-Hong Wang, Bing-You Yang, Zhi-Bin Wang, Yong-Gang Xia

**Affiliations:** Key Laboratory of Chinese Materia Medica, Heilongjiang University of Chinese Medicine, Ministry of Education, Harbin 150040, China

**Keywords:** *Sanguisorba tenuifolia* var. Alba, triterpenoids, α-glucosidase inhibitory activity, diabetes mellitus

## Abstract

The ethyl acetate soluble fraction from the roots of *Sanguisorba tenuifolia* was found to have a hypoglucemic effect in alloxan-induced diabetic rats. Two new triterpenoids, identified as 2-oxo-3*β*,19*α*-dihydroxyolean-12-en-28-oic acid *β*-D-gluco-pyranosyl ester (**1**) and 2*α*,19*α*-dihydroxy-3-oxo-12-ursen-28-oic acid *β*-D-glucopyranosyl ester (**4**) were isolated from this fraction, along with thirteen known triterpenoids. Their structures were elucidated by chemical and spectroscopic methods. All these compounds demonstrated inhibitory activities against *α*-glucosidase with IC_50_ values in the 0.62-3.62 mM range.

## 1. Introduction

Diabetes mellitus (DM), considered a lifestyle related diseases, is a metabolic disease with hyper-glycemia as a symptom and causes many complications [1]. Recently, DM is becoming a serious problem around the World, and according to World Health Organization, it affects approximately 171 million people worldwide and the number is expected to reach to 366 million over the next 20 years [2]. Many researchers have enthusiastically studied the development of antidiabetic agents, however, many potential therapeutics have a number of serious adverse effects [3,4], therefore there is a growing trend toward using natural products as treatment [5]. China has a long history of using herbs for the treatment of human diseases and several medicinal plants are used for the treatment of diabetes. *S. tenuifolia* is one such plant.

**Figure 1:**
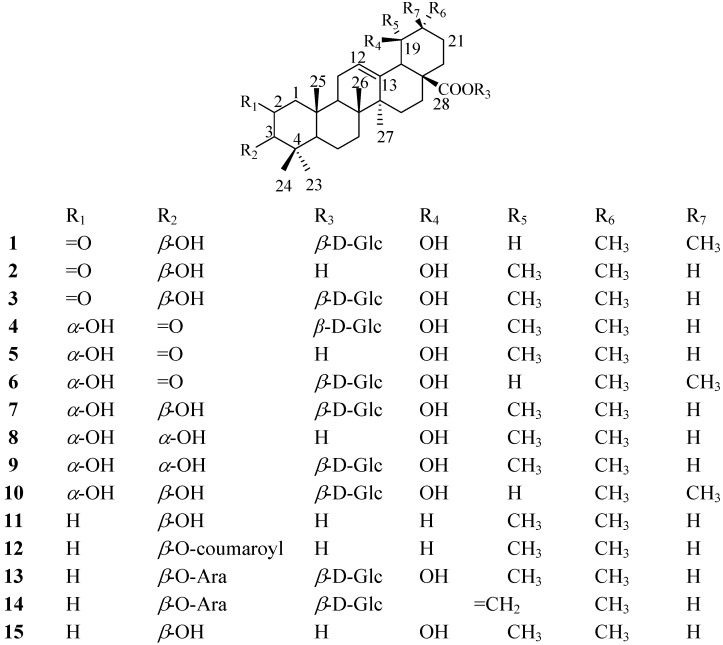
Structures of **1**-**15**.

*S. tenuifolia* (Rosaceae) is a perennial herb, which is widely distributed in China’s Heilongjiang, Liaoning, and Jilin provinces and Inner Mongolia. The residents in Northeast China regard *S. tenuifolia* as a substitute for *S. officinalis*, and apply its roots for the treatment of diarrhea, chronic intestinal infections, duodenal ulcers, diabetes mellitus and bleeding [6,7]. Our studies indicated that ethyl acetate fraction of *a* *S. tenuifolia* root ethanol extract contains plenty of triterpenes, which can inhibit plasma glucose levels in alloxan-induced diabetic rats. *α*-Glucosidase inhibitors are oral anti-diabetic drugs used for diabetes mellitus type 2. They can significantly delay the absorption of carbohydrates from the small intestine and thus have a lowering effect on postprandial blood glucose and insulin levels [8]. Based on a bioassay-guided isolation, a phytochemical study of *S. tenuifolia* was performed and two new triterpenoids were isolated from its ethyl acetate fraction, along with thirteen other known triterpenoids. The new compounds were identified as 2-oxo-3*β*,19*α*-dihydroxy-olean-12-en-28-oic acid *β*-D-glucopyranosyl ester (**1**) and 2*α*,19*α*-dihydroxy-3-oxo-12-ursen-28-oic acid *β*-D-glucopyranosyl ester (**4**), respectively. In the present report, we describe the structural elucidation of **1** and **4**, together with the *α*-glucosidase inhibitory activity data of all the compounds **1**-**15** (Figure 1). 

## 2. Results and Discussion

Compound **1** was obtained as a white amorphous powder. The HR-ESI-MS data indicated a molecular formula of C_36_H_56_O_10_, based on the [*M* + H]^+^ ion signal at *m/z* 649.3953 (calc. C_36_H_57_O_10_, 649.3952), [*M* + NH_4_]^+^ ion signal at *m/z* 666.4221 (calc. C_36_H_60_O_10_N, 666.4217) and [*M* + Na]^+^ ion signal at *m/z* 671.3779 (calc. C_36_H_56_O_10_Na, 671.3771). The IR spectrum showed the presence of hydroxyl groups (3,419.6 cm^−1^), ester carbonyl (1,735.3 cm^−1^), carbonyl (1,711.3 cm^−1^) and double bond (1,640.8 cm^−1^). 

**Figure 2:**
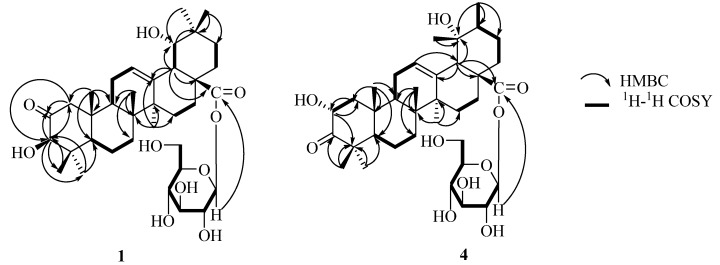
Key HMBC and ^1^H-^1^H COSY correlations of **1** and **4**.

The ^13^C-NMR spectrum and DEPT of **1** showed seven methyl, nine methylene, eleven methine, and nine quaternary carbon signals, including one ester carbonyl at *δ_C_* 177.3, a quaternary olefinic carbonyl at *δ_C_* 144.5, one anomeric carbon signal at *δ_C_* 95.9, a ketone carbonyl at*δ_C_* 213.4. The ^1^H-NMR spectrum exhibited seven singlet methyl signals at *δ_H_* 1.21, 0.92, 1.01, 1.15, 1.52, 1.12 and 0.95, an anomeric proton signal at *δ_H_* 6.37 (d, *J* = 8.0 Hz), two methine proton signals at *δ_H_* 3.89 (s) and 3.54 (d, *J* = 2.8 Hz), and an olefinic proton signal at *δ_H_* 5.45 (br s), which were characteristic of the oleanolic acid skeleton. Comparison of the data **1** with those of oleanolic acid [9,10], suggested that the aglycone of **1** was an oleanolic acid derivative with one hydroxyl group at the ring E portion, as well as one ketone carbonyl group. The proton signal at*δ_H_* 3.50 showed long-rang correlations with C-13, C-17, and C-28 in the HMBC spectrum, and was assigned to the H-18 (Figure 2). This proton had a proton spin-coupling correlation with the signal at *δ_H_* 3.54, which was associated with the carbon signal at *δ_C_* 81.0 (CH) in the HSQC spectrum. Thus, the presence of a hydroxyl group at C-19 was evident. The ^3^*J*_H,H_ value of 2.8 Hz between H-18 and H-19, and NOE correlations from H-19 to Me-29 and Me-30 gave evidence for the C-19*α* hydroxy orientation [11]. There were long-range correlations between protons and carbons: H-3 (*δ_H_* 3.89)/C-23 (*δ_C_* 27.6), C-24 (*δ_C_* 21.7), and ketone carbonyl (*δ_C_* 213.4); H-23 (*δ_H_* 1.21), H-24 (*δ_H_* 0.92)/C-3 (*δ_C_* 83.2) in the HMBC spectrum, which indicated that the ketone carbonyl group must be either at position C-1 or C-2. Furthermore, the long-range correlations were observed between protons and carbons: H-3 (*δ_H_* 3.89), H-25 (*δ_H_* 1.01)/C-1 (*δ_C_* 51.5), H-1 (*δ_H_* 3.00, 2.27)/ketone carbonyl (*δ_C_* 213.4) in the HMBC spectrum (Figure 2). The ketone must be at the C-2 position based on comparison of the NMR spectral data for C-1, C-2 and C-3 of **1** with that of the similar compound 2-oxopomolic acid (**2**) [C-1 (*δ_C_* 53.6 *δ_H_* 2.46, 2.15), C-2 (*δ_C_* 211.2) and C-3 (*δ_C_* 83.5; *δ_H_* 4.17 s)] and 2*α*,19*α*-dihydroxy-3-oxo-olean-12-en-28-oic acid *β*-D-glucopyranosyl ester (**6**) [C-2 (*δ_C_* 216.5) and C-3 (*δ_C_* 69.7; *δ_H_* 4.80 s)] [12,13]. Based on these findings, the structure of the aglycone part of **1** was established to be 2-oxo-3*β*,19*α*-dihydroxyolean-12-en-28-oic acid, a new triterpene. The configuration of the sugar unit was assigned after hydrolysis of **1** with 1 M HCl. The acid hydrolysis gave D-glucose. The data of anomeric carbon signal at *δ_C_* 95.9 and anomeric proton signal at *δ_H_* 6.37 (d, *J* = 8.0 Hz) indicated the glucose was in the *β* form and was bound to the aglycone by a glycosidic linkage at C-28 in the HMBC spectrum (Figure 2). Therefore, the structure of compound **1 **was elucidated as 2-oxo-3*β*,19*α*-dihydroxy-olean-12-en-28- oic acid *β*-D-glucopyranosyl ester.

Compound **4** was obtained as a white amorphous powder. The HR-ESI-MS data indicated a molecular formula of C_36_H_56_O_10_based on the [*M* + H]^+^ ion signal at *m/z* 649.3945 (calc. 649.3952) in the. The IR spectrum showed the presence of hydroxyl groups (3431.1 cm^−1^), ester carbonyl (1,737.1 cm^−1^), carbonyl (1,714.3cm^−1^), and double bond (1,644.6 cm^−1^).

The ^13^C-NMR spectrum shows seven methyl, nine methylene, eleven methine, and nine quaternary carbon signals, including one ester carbonyl at *δ_C_* 177.0, a quaternary olefinic carbonyl at *δ_C_* 139.5, one anomeric carbon signal at *δ_C_* 95.9, a ketone carbonyl at*δ_C_* 216.6. Its ^1^H-NMR spectrum shows the presence of a hydroxymethine proton at *δ_H_* 4.82 (1H, dd, *J* = 12.5, 6.3 Hz), one trisubstituted olefinic proton at (*δ_H_* 5.50, br s), six singlets at *δ_H_* 1.19, 0.99, 1.18, 1.15, 1.59, 1.37 for six tertiary methyl groups, one secondary methyl group (*δ_H_* 1.05, d, *J* = 6.6Hz), one methine proton characteristic of H-18 of pomolic acid (*δ_H_* 2.91, s), and one anomeric proton (*δ_H_* 6.30 d, *J* = 8.0 Hz). The secondary methyl signal on ring E provides a most useful indicator for the presence of an urs-12-ene skeleton [10]. Additionally, the signals in its ^13^C-NMR at *δ_C_* 128.0 and 139.5 are characteristic for a C-12/C-13 double bond in the ursene-type structure [14]. Acid hydrolysis of **4** with 1 M HCl (5 mL) gave a D-glucose molecule and a triterpene (C_30_H_46_O_5_, **5**). The latter was identified as 2*α*,19*α*-dihydroxy-3-oxo-12-ursen-28-oic acid by comparing its spectral and physical data with literature values [15]. When the ^1^H- and ^13^C-NMR spectra of **4 **were compared with those of **5**, an upfield shift due to the glycoside was detected at the C-28 signal at *δ_C_* 177.0. The linked site of glycosyl group in **4** was further established from correlations between the anomeric proton H-1' at *δ_H_* 6.30 and C-28 at *δ_C_* 177.0 in the HMBC spectrum (Figure 2). Therefore, the structure of **4** was determined as 2*α*,19*α*-dihydroxy-3-oxo-12-ursen-28-oic acid *β*-D-glucopyranosyl ester.

**Table 1:** NMR data of **1** and **4** in pyridine-*d_5_* (*δ* in ppm, *J* in Hz, recorded at 400 MHz and 100 MHz, respectively).

No.	1		4	
	*δ_C_* (DEPT)	*δ_H_* (*J*, Hz)	*δ_C_* (DEPT)	*δ_H_* (*J*, Hz)
1	51.5 (CH_2_)	3.00 d (12.4 ), 2.27 d (12.4)	50.3 (CH_2_)	2.48 dd (12.5, 6.3), 1.37 m
2	213.4 (C)		69.8 (CH)	4.82 dd (12.5, 6.3)
3	83.2 (CH)	3.89 s	216.6 (C)	
4	42.2 (C)		48.2 (C)	
5	50.2 (CH)	2.02 m	57.7 (CH)	1.23 m
6	19.3 (CH_2_)	1.46 m, 1.31 m	19.6 (CH_2_)	1.34 m, 1.29 m
7	33.2 (CH_2_)	1.43 m, 1.70 m	33.2 (CH_2_)	1.43 m, 1.33 m
8	40.3 (C)		40.6 (C)	
9	48.1 (CH)	1.83 m	47.4 (CH)	1.83 m
10	42.8 (C)		37.8 (C)	
11	24.3 (CH_2_)	2.02 m	24.2 (CH_2_)	2.08 m
12	123.0 (CH)	5.45 br s	128.0 (CH)	5.50 br s
13	144.5 (C)		139.5 (C)	
14	42.3 (C)		42.2 (C)	
15	29.0 (CH_2_)	2.46 m, 1.20 m	29.2 (CH_2_)	2.49 m, 1.22 m
16	27.9 (CH_2_)	2.81 m, 2.12 m	26.1 (CH_2_)	3.09 m, 2.05 m
17	46.5 (C)		48.6 (C)	
18	44.6 (CH)	3.50 d (2.8)	54.4 (CH)	2.91 s
19	81.0 (CH)	3.54 d (2.8)	72.7 (CH)	
20	35.6 (CH)		42.2 (CH)	1.39 m
21	29.0 (CH_2_)	1.24 m, 2.35 m	26.7 (CH_2_)	1.24 m, 2.02 m
22	33.0 (CH_2_)	2.04 m, 1.93 m	37.9 (CH_2_)	2.03 m, 1.83 m
23	27.6 (CH_3_)	1.21 s	25.4 (CH_3_)	1.19 s
24	21.7 (CH_3_)	0.92 s	21.8 (CH_3_)	0.99 s
25	16.9 (CH_3_)	1.01 s	17.6 (CH_3_)	1.18 s
26	17.1 (CH_3_)	1.15 s	16.1 (CH_3_)	1.15 s
27	24.9 (CH_3_)	1.52 s	24.6 (CH_3_)	1.59 s
28	177.3 (C)		177.0 (C)	
29	28.7 (CH_3_)	1.12 s	27.0 (CH_3_)	1.37 s
30	24.4 (CH_3_)	0.95 s	16.8 (CH_3_)	1.05 d (6.6)
1'	95.9 (CH)	6.37 d (8.0)	95.9 (CH)	6.30 d (8.0)
2'	74.1 (CH)	4.22 t (8.4)	74.1 (CH)	4.24 t (8.4)
3'	79.3 (CH)	4.29 t (8.8)	79.4 (CH)	4.34 t (8.7)
4'	71.2 (CH)	4.38 t (9.0)	71.2 (CH)	4.39 t (9.3)
5'	79.0 (CH)	4.04 m	79.0 (CH)	4.05 (m)
6'	62.2 (CH_2_)	4.42 br d (12.1), 4.46 dd (12.1, 3.8)	62.4 (CH_2_)	4.42 br d (11.7), 4.49 dd (11.7, 4.4)

The other compounds were characterized as 2-oxopomolic acid (**2**) [12], 2-oxopomolic acid *β*-D-glucopyranoside (**3**) [12], 2*α*,19*α*-dihydroxy-3-oxo-12-ursen-28-oic acid (**5**) [15], 2*α*,19*α*-dihydroxy-3-oxo-olean-12-en-28-oic acid *β*-D-glucopyranosyl ester (**6**) [13], rosamutin (**7**) [16], euscaphic acid (**8**) [17], kaji-ichigoside Fl (**9**) [16], 24-deoxysericoside (**10**) [17], ursolic acid (**11**) [10], *p*-coumaroylursolic acid (**12**) [18], ziyu-glycoside I (**13**) [17], 3*β*-[(*α*-L-arabinopyranosyl) oxy] urs-12,19(29)-dien-28-oic acid 28-*β*-D-glucopyranosyl ester (**14**) [11] and pomolic acid (**15**) [17] by comparing their NMR spectroscopic data with the literature values. All these known compounds are reported for the first time in *S. tenuifolia*.

We next evaluated the isolated compounds for their inhibitory activity against *α*-glucosidase since some compounds are known *α*-glucosidase inhibitors [19]. The results are shown in Table 2, with acarbose used as a positive control. Compounds **1**-**15** exhibited dose-dependent *α*-glucosidase inhibitory activities with IC_50_ values of 0.62-3.62 mM. Compounds **8** and **12** showed the most potent activity (IC_50_ 0.67 and 0.62 mM, respectively), comparable with the positive control. Triterpenoids of the EtOAc-soluble fraction may be the potential anti-hypoglycemic agents in this plant, as they have been shown to induce an anti-diabetic effect. 

**Table 2:** *In vito* α-glucosidase inhibitory assay.

Compound	IC_50 _(mM ± SEM, mM)
**1**	1.88 ± 0.28
**2**	1.35 ± 0.04
**3**	2.22 ± 0.06
**4**	1.56 ± 0.04
**5**	1.23 ± 0.09
**6**	2.01 ± 0.06
**7**	3.28 ± 0.08
**8**	0.67 ± 0.09
**9**	3.10 ± 0.24
**10**	3.52 ± 0.16
**11**	1.69 ± 0.04
**12**	0.62 ± 0.06
**13**	3.62 ± 0.21
**14**	2.87 ± 0.06
**15**	1.84 ± 0.12
Acarbose	0.79 ± 0.13

## 3. Experimental Section

### 3.1. General

Open column chromatography (CC) was carried out using silica gel (200-300 mesh, Qingdao Marine Chemical Co., Qingdao, China) or octadecyl silica gel (ODS, 25-40 μm, Fuji) as stationary phases. TLC employed precoated silica gel plates (5-7 μm, Qingdao Marine). Preparative HPLC was carried out on a Waters 600 instument equipped with a Waters UV-2487 detector. A Waters Sunfire prep C18 OBD (19 × 250 mm i.d.) column was used for this purpose. The IR spectra were recorded as KBr pellets on a Jasco 302-A spectrometer. Optical rotation was recorded on a Jasco P-2000 polarimeter. HRESIMS were measured on a FTMS-7 instrument (Bruker Daltonics). Melting points were determined on a Gallenkemp apparatus and are uncorrected. The ^1^H-, ^13^C- and 2D (^1^H-^1^H COSY, HSQC, HMBC, NOESY) NMR spectra were recorded on a Bruker AMX-400 spectrometer using standard pulse sequences. Chemical shifts are reported in ppm (*δ*), and scalar coupling are reported in Hz. GC analyses were carried out using a Fuli 9790 instrument equipped with a DM-5 column (0.25 μm, 30 m × 0.25 mm, Dikma, China). *α*-Glucosidase (EC.3.2.1.20) from *Saccharomyces* sp. was purchased from Wako Pure Chemical Indutries Ltd. (Wako 076-02841). Other reagents were purchased from various commercial sources.

### 3.2. Plant Material

The roots of *S. tenuifolia* were collected in October 2008 from Fangzheng of Heilongjiang Province, China, and identified by Zhenyue Wang, of Heilongjiang University of Chinese Medicine. A voucher specimen (20081023) was deposited at the herbarium of Heilongjiang University of Chinese Medicine, Harbin, China.

### 3.3. Extraction and Isolation

The dried roots of *S. tenuifolia* (5.0 kg) were extracted with 70% EtOH (3 × 10 L) to afford the EtOH extract (1.3 kg) which was then suspended in water (10 L) and then extracted with petroleum ether and ethyl acetate (EtOAc) (3 × 10 L each), yielding petroleum ether (10.2 g) and ethyl acetate (222.5 g) extracts. The EtOAc fraction (222.5 g) was subjected to silica gel column with a stepwise CH_2_Cl_2_-MeOH gradient (30:1; 20:1; 10:1; 5:1, v/v), and finally with MeOH alone, to give five fractions I-V. Fraction I (40.8 g) was separated using silica gel CC eluting with CH_2_Cl_2_-MeOH (50:1, 30:1, 10:1, v/v) to obtain three sub-fractions, I_1_-I_3_. Sub-fraction I_2 _(10.6 g) was further separated by ODS silica gel CC with MeOH-H_2_O (9:1, v/v) and to **11** (33.2 mg), **12** (37.5 mg) and **15** (25.5 mg); Fraction II (38.3 g) was subjected to silica gel CC eluting with CH_2_Cl_2_-MeOH (30:1, 20:1, 10:1, v/v) to afford four sub-fractions, II_1_-II_4_. Sub-fraction II_1_ (13.3 g) afforded compounds **2** (21.0 mg), **5** (44.5 mg) and **8** (24.6 mg, *t*_R_ = 50.5 min), after subjecting it to ODS silica gel CC eluting with MeOH-H_2_O (3:1, 3:2, v/v), followed by preparative HPLC with MeOH-H_2_O (4:1, v/v).Fraction III (31.3 g) was subjected to silica gel CC eluting with CH_2_Cl_2_-MeOH (8:1, 5:1, 1:1, v/v) to afford four sub-fractions, III_1_-III_4_. Sub-fraction III_2 _(7.3 g) afforded **4** (43.5 mg) and a mixture of **1**, **3** and **6** by ODS silica gel CC using MeOH-H_2_O (2:1, v/v) as eluent. The mixture was separated into **1** (25.2 mg, *t*_R_ = 45.3 min), **3** (23.4 mg *t*_R_ = 43.2 min) and **6** (12.5 mg, *t*_R_ = 48.5 min) by preparative HPLC using MeOH-H_2_O (3:2, v/v). Fraction IV (43.1 g) was applied to a silica gel column which was eluted with CH_2_Cl_2_-MeOH-H_2_O (8:1:0.1, 6:1:0.1, 3:1:0.1, v/v) to afford four sub-fractions, IV_1_-IV_4_. Sub-fraction IV_2_ (16.3 g) afforded a mixture of **7**, **9** and **10**, along with a few impurities, after ODS silica gel CC with MeOH-H_2_O (2:1, v/v). The mixture was separated by preparative HPLC using MeOH-H_2_O (3:2, v/v) into **7** (27.5 mg, *t*_R_ = 49.2 min), **9** (25.2 mg, *t*_R_ = 43.2 min) and **10** (30.4 mg, *t*_R_ = 45.2 min). Fraction V (40.1 g) was applied to a silica gel column eluted with CH_2_Cl_2_-MeOH-H_2_O (6:1:0.1, 3:1:0.1, v/v) to afford three sub-fractions, V_1_-V_2_. Sub-fraction V_1_ (13.3 g) afforded **13** (60.8 mg) and **14** (19.4 mg) by ODS silica gel CC eluting with MeOH-H_2_O (2:1, v/v). 

*2-Oxo-3β,19α-dihydroxyolean-12-en-28-oic acid* *β-D-glucopyranosyl ester* (**1**). White amorphous powder. 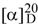 + 16.5° (*c* 1.05, MeOH). IR (KBr): 3419.6, 1735.7, 1711.3, 1640.8, 1070.4, 1029.9, 993.3 cm^-1^. HR-ESI-MS *m/z* 671.3779 [*M* + Na]^+^ (calc. C_36_H_56_O_10_Na, 671.3771), 649.3953 [*M* + H]^+^ (calc. C_36_H_5__7_O_10_, 649.3952), 666.4221 [*M* + NH_4_]^+^ (calc. C_36_H_60_O_10_N, 666.4217); ^1^H- and ^13^C-NMR (pyridine-*d_5_*) data, see Table 1.

2*α*,19*α*-Dihydroxy-3-oxo-12-ursen-28-oic acid *β*-D-glucopyranosyl ester (**4**). White amorphous powder. 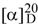 + 21.5° (*c* 1.25, MeOH). IR (KBr): 3431.1, 1737.1, 1714.3, 1644.6, 1070.4, 1029.9, 991.3 cm^-1^. HR-ESI-MS *m/z* 649.3945 [*M* + H]^+^ (calc. C_36_H_5__7_O_10_, 649.3952); ^1^H- and ^13^C-NMR (pyridine-*d_5_*) data, see Table 1.

#### 3.3.1. Acid Hydrolysis of **1** and **4** and Determination of the Absolute Configuration of the Mono-saccharides

**1** (5 mg) in 1 M HCl (dioxane-H_2_O, 1:1, 5 mL) was heated at 90 °C for 3 h under an Ar atmosphere. After the dioxane was removed, the solution was extracted with EtOAc (3 mL × 3) to remove the aglycone. The aqueous layer was neutralized by passing through an ion-exchange resin column (Amberlite MB-3, Organo, Tokyo, Japan) and concentrated to dryness under reduced pressure to give the sugar fraction. The residue was dissolved in pyridine (0.1 mL) to which 0.1 M L-cysteine methyl ester hydrochloride in pyridine (0.1 mL) was added. The mixture was heated at 60 °C for 1 h. After the reaction mixture was dried in vacuo, the residue was trimethylsilylated with l-trimethylsilylimidazole (0.2 mL) for 2 h. The mixture was partitioned between hexane and H_2_O (0.6 mL, each), and the hexane extracted was analyzed by GC under the following conditions: capillary column, DM-5 (0.25 mm × 30 m × 0.25 μm); detector, FID; injector temperature, 280 °C, detector temperature, 280 °C; initial temperature was maintained at 160 °C for 2 min and then raised to 195 °C at a rate of 10 °C/min; carrier gas, N_2_. In the acid hydrolysate of **1**, D-glucose was confirmed by comparison of the retention time of their derivatives with those of D-glucose and L-glucose derivatives prepared in a similar way, which showed retention times of 28.56 and 27.72 min, respectively. The sugar from **4** (30 mg) was also identified by the same method.

#### 3.3.2. α-Glucosidase Inhibition Assay

*α*-Glucosidase (EC.3.2.1.20) enzyme inhibition assay has been performed according to the literature [19]. *α*-Glucosidase (25 μL, 0.2 U/mL), various concentrations of samples (25 μL), and 67 mM phosphate buffer (pH 6.8, 175 μL) were mixed at room temperature for 10 min. Reactions were initiated by the addition of 23.2 mM *p*-nitrophenyl-*α*-D-glucopyranoside (25 μL). The reaction mixtures were incubated at 37 °C for 15 min in a final volume of 250 μL, and then 1 M Na_2_CO_3_ (50 μL) was added to the incubation solution to stop the reaction. The activities of glucosidase were detected in a 96-well plate, and the absorbance was read at 405 nm by a microplate spectrophotometer (Spectra Max, Molecular Devices, USA). The negative control was prepared by adding phosphate buffer instead of the sample in the same way as the test. Acarbose was utilized as the positive control. The blank was prepared by adding phosphate buffer instead of *α*-glucosidase using the same method. The inhibition rates (%) were calculated from the following formula: 

[(OD_negative control_− OD_blank_) − (OD_test_− OD_test blank_)] / (OD_negative blank_− OD_blank_) × 100%

## 4. Conclusions

Two new triterpenoids, 2-oxo-3*β*,19*α*-dihydroxyolean-12-en-28-oic acid *β*-D-glucopyranosyl ester (**1**) and 2*α*,19*α*-dihydroxy-3-oxo-12-ursen-28-oic acid *β*-D-glucopyranosyl ester (**4**) were isolated from an ethyl acetate fraction of* S. tenuifolia* roots, along with thirteen known triterpenoids. All these triterpenoids are reported for the first time in *S. tenuifolia* and demonstrated inhibitory activities against *α*-glucosidase with IC_50_ values in the 0.62-3.62 mM range. Triterpenoids of the EtOAc-soluble fraction may be the potential anti-hypoglycemic agents in this plant, as they have been shown to induce an anti-diabetic effect.

## References

[1] Rasmussen L.M., Ledet T. (1996). Aortic atherosclerosis in diabetes mellitus is associated with an insertion/deletion polymorphism in the angiotensin I-converting enzyme gene. No relation between the polymorphism and aortic collagen content. Diabetologia.

[2] World Health Organization Country and regional data. http://www.who.int/diabetes/facts/world_figures/en/.

[3] Bhatnagar D. (1998). Lipid-lowering drugs in the management of hyperlipidemia. Pharmacol. Therapeut..

[4] May L., Lefkowitch J., Kram M., Rubin D. (2002). Mixed hepatocelluar-cholestatic liver injury after pioglitazone therapy. Ann. Intern. Med..

[5] Jung M., Park M., Lee H.C., Kang Y.H., Kang E.S., Kim S.K. (2006). Antidiabetic agents from medicinal plants. Curr. Med. Chem..

[6] Zhu Y.C. (1998). Plantae Medicinal Chinae Boreali-Orientalis.

[7] Jiangsu New Medical College (1997). The Chinese Medicine Dictionary.

[8] Sou S., Mayumi S., Takahashi H., Yamasaki R., Kadoya S., Sodeoka M., Hashimoto Y. (2000). Novel alpha-glucosidase inhibitors with a tetrachlorophthalimide skeleton. Bioorg. Med. Chem. Lett..

[9] Kizu H., Shimana H., Tomimori T. (1995). Studies on the constituents of Clematis species. VI1. The constituents of *Clematis stans* SIEB. et ZUCC. Chem. Pharm. Bull..

[10] Reher G., Budesinsky M. (1992). Triterpenoids from plants of the Sanguisorbeae. Phytochemistry.

[11] Mimaki Y., Fukushima M., Yokosuka A., Sashida Y., Furuya S., Sakagami H. (2001). Triterpene glycosides from the roots of *Sanguisorba officinalis*. Phytochemistry.

[12] Jia Z.J., Liu X.Q., Liu Z.M. (1993). Triterpenoids from *Sanguisorba alpina*. Phytochemistry.

[13] Chouksey B.K., Srivastava S.K. (2001). New constituent from the roots of *Terminalia arjuna*: Antifungal agent. Indian J. Chem. Sect. B: Org. Chem. Incl. Med. Chem..

[14] Durham D.G., Liu X., Richards R.M. (1994). A triterpene from *Rubus pinfaensis*. Phytochemistry.

[15] Xu H.X., Zeng F.Q., Wan M., Sim K.Y. (1996). Anti-HIV triterpene acids from *Geum japonicum*. J.Nat.Prod..

[16] Zhou X.H., Kasai R., Ohtani K., Tanaka O., Nie R.L., Yang C.R., Zhou J., Yamasaki K. (1992). Oleanane and ursane glucosides from *Rubus* species. Phytochemistry.

[17] Cheng D.L., Cao X.P. (1992). Pomolic acid derivatives from the root of *Sanguisorba officinalis*. Phytochemistry.

[18] Tanachatchairatana T., Bremner B.J., Chokchaisiri R., Suksamrarn A. (2008). Antimycobacterial activity of cinnamate-based esters of the triterpenes betulinic, oleanolic and ursolic acids. Chem. Pharm. Bull..

[19] Li W., Fu H.W., Bai H., Sasaki T., Kato H., Koike K. (2009). Triterpenoid saponins from *Rubus ellipticus* var. *obcordatus*. J. Nat. Prod..

